# Glomerular filtration rate in patients with atrial fibrillation and 1-year outcomes

**DOI:** 10.1038/srep30271

**Published:** 2016-07-28

**Authors:** Giuseppe Boriani, Cécile Laroche, Igor Diemberger, Mircea Ioachim Popescu, Lars Hvilsted Rasmussen, Lucian Petrescu, Harry J. G. M. Crijns, Luigi Tavazzi, Aldo P. Maggioni, Gregory Y. H. Lip

**Affiliations:** 1 Cardiology Department, University of Modena and Reggio Emilia, Policlinico di Modena, Modena, Italy; 2 Institute of Cardiology, Department of Experimental, Diagnostic and Specialty Medicine, University of Bologna, S.Orsola-Malpighi University Hospital, Bologna, Italy; 3 EURObservational Research Programme Department, European Society of Cardiology, Sophia Antipolis, France; 4 Faculty of Medicine, Cardiology Department, Oradea, Romania; 5 Department of Cardiology, Aalborg University Hospital and Aalborg Thrombosis Research Unit, Department of Clinical Medicine, Faculty of Medicine Aalborg University, Aalborg, Denmark; 6 Institute of Cardio-vascular Diseases, University of Medicine and Pharmacy “Victor Babes”, Timisoara, Romania; 7 Department of Cardiology and Cardiovascular Research Institute Maastricht (CARIM), Maastricht University Medical Center, The Netherlands; 8 Maria Cecilia Hospital, GVM Care & Research. E.S. Health Science Foundation, Cotignola, Italy; 9 ANMCO Research Center, Firenze Italy; 10 University of Birmingham Centre for Cardiovascular Sciences, City Hospital, Birmingham B18 7QH, United Kingdom

## Abstract

We assessed 1-year outcomes in patients with atrial fibrillation enrolled in the EurObservational Research Programme AF General Pilot Registry (EORP-AF), in relation to kidney function, as assessed by glomerular filtration rate (eGFR). In a cohort of 2398 patients (median age 69 years; 61% male), eGFR (ml/min/1.73 m^2^) calculated using the CKD-EPI formula was ≥80 in 35.1%, 50–79 in 47.2%, 30–49 in 13.9% and <30 in 3.7% of patients. In a logistic regression analysis, eGFR category was an independent predictor of stroke/TIA or death, with elevated odds ratios associated with severe to mild renal impairment, ie. eGFR < 30 ml/min/1.73 m^2^ [OR 3.641, 95% CI 1.572–8.433, p < 0.0001], 30–49 ml/min/1.73 m^2^ [OR 3.303, 95% CI 1.740–6.270, p = 0.0026] or 50–79 ml/min/1.73 m2 [OR 2.094, 95% CI 1.194–3.672, p = 0.0003]. The discriminant capability for the risk of death was tested among various eGFR calculation algorithms: the best was the Cockcroft-Gault equation adjusted for BSA, followed by Cockcroft-Gault equation, and CKD-EPI equation, while the worst was the MDRD equation. In conclusion in this prospective observational registry, renal function was a major determinant of adverse outcomes at 1 year, and even mild or moderate renal impairments were associated with an increased risk of stroke/TIA/death.

Atrial fibrillation (AF) is the most common sustained arrhythmia, and its incidence and prevalence are increasing worldwide^1^. Given its close association with age and various comorbidities, AF is commonly associated with impairment in renal function, of various degrees^2^.

AF is associated with an increased risk of stroke and thromboembolic events and risk stratification is essential for appropriate decision making with regard to anticoagulants^3^,^4^. There is growing interest on assessing renal function in patients with AF since compromise of renal function has major implications with regard to the risk of stroke and bleeding^5^. Indeed, a precise estimate of renal function is necessary in patients with non-valvular AF^6^,^7^ who are candidates for treatment with the non vitamin K antagonist oral anticoagulants (NOACs)^8^.

Chronic kidney disease (CKD) is defined as the presence of kidney abnormalities, which can involve its structure and/or its function, for a period longer than 3 months, and glomerular filtration rate (GFR) is widely accepted as the best overall index of kidney function^2^. The GFR can be estimated from the serum creatinine using a number of different equations to give an estimated GFR (eGFR)^2^,^9^. Clinical Practice Guidelines delivered by KDIGO (Kidney Disease: Improving Global Outcomes) group for the evaluation and management of CKD recommended, in 2012, use of the CKD EPI equation for estimation of eGFR, on the basis of standardized serum creatinine, and for staging of kidney function impairment^2^,^9^. This recommendation is not concordant with the advice to use Cockcroft-Gault equation for evaluating kidney function for the prescription of NOACs in cardiology practice^8^.

The objective of this report from the EURObservational Research Programme – Atrial Fibrillation (EORP-AF) General Pilot Registry was to investigate the baseline characteristics and the outcomes at 1 year follow-up of prospectively enrolled AF patients presenting to cardiologists, in relation to kidney function, as assessed by different equations for estimated glomerular filtration rate (eGFR) and 1-year outcomes, in terms of stroke and mortality in European AF patients followed by cardiologists. The analysis had also the aim to assess the concordance between the different equations proposed for estimating GFR and the potential differences in terms of outcome prediction.

The EORP-AF General Pilot Registry^10^,^11^,^12^,^13^,^14^,^15^ is a multicenter European registry which enrolled consecutive in- and out-patients presenting with documented AF to cardiologists, in participating centres from 9 European countries and includes patients with both valvular and non valvular AF with no exclusion criteria on the basis of co-morbidities such as renal or hepatic impairment. An additional objective of our analysis was to compare 4 different equations of common clinical use for calculating eGFR with regard to concordance in estimate of kidney function and in terms of outcome prediction.

## Methods

The methods and baseline data from the EORP-AF pilot general registry have previously been published^8^. The registry was commenced in early 2012. One-year follow-up phase (‘pilot phase’ or Phase 1) data were focused on patients from 9 countries (for a broad representation of European Society of Cardiology member countries) recruited into this database^13^.

In brief, the registry population comprised consecutive in- and out-patients presenting with AF to cardiologists, enrolled in 67 centres in 9 countries^10^,^11^,^12^,^13^,^14^,^15^. Consecutive patients were screened at the time of their presentation to a cardiologist (hospital or medical centre), and potential patients were approached to obtain written informed consent according to local rules. The protocol of EORP AF was initially approved by the European Society of Cardiology (Sophia Antipolis, France) and then by the Institutional Review Boards, at a national or local level, according to country regulations. The research was performed in accordance with the ethical standards laid down in the 1964 Declaration of Helsinki and its later amendments. Enrollment required ECG-confirmed diagnosis of AF, with a qualifying episode of AF documented in the 12 months prior to enrollment. Stroke risk was categorised using the CHA_2_DS_2_-VASc score^10^,^11^,^12^,^13^,^14^,^15^, whilst bleeding risk was categorised using the HAS-BLED score^10^,^11^,^12^.

In this registry, parameters collected at enrollment included history of CKD, serum creatinine, body weight and the other parameters that permitted us to calculate eGFR according to the CKD Epidemiology Collaboration (CKD-EPI) equation, MDRD equation, Cockcroft-Gault equation, and BSA adjusted- Cockcroft-Gault equation^2^ (Table w1, web only appendix). Patients were classified on the basis of eGFR in 4 groups, corresponding to eGFR ≥80 ml/min/1.73 m^2^, between 50 and 79 ml/min/1.73 m^2^, between 30 and 49 ml/min/1.73 m^2^ and <30 ml/min/1.73 m^2^, respectively. The choice of the cuf-offs was in line with the cut-off commonly used for prescribing the appropriate dose of NOACs^8^. For the main analysis, patient classification according to eGFR calculated with CKD-EPI equation was used.

Outcomes were recorded for all cause mortality, cardiovascular death, thromboembolism (TE) and bleeding. TE refers to stroke, transient ischaemic attack (TIA), acute coronary syndrome (ACS), coronary intervention, cardiac arrest, peripheral embolism and pulmonary embolism – each of these were as recorded by the investigator, in this ‘real world’ observational registry.

### Statistical analyses

Univariate analysis was applied to both continuous and categorical variables. Continuous variables were reported as mean ± SD and/or as median and Interquartile Range (IQR), as appropriate. Among-group comparisons were made using a non-parametric test (Kruskal-Wallis test). Categorical variables were reported as percentages. Among-group comparisons were made using a Chi-square test or a Fisher’s exact test if any expected cell count was less than five.

The association between eGFR and stroke/TIA or death at 1 year was analysed by logistic models. Odds ratios of eGFR with CKD-EPI equation were obtained with different models.

The first model included age and sex. The second model included age, sex and co-morbidities like coronary artery disease, chronic heart failure, previous stroke, previous TIA, ischaemic thrombo-embolic complications, haemorrhagic events and malignancy. At the second model, were added in the third model other potential confounding factors (variables with p < 0.10 in univariate, except those with a high number of missing data). And finally, among the last confounding factors a stepwise multiple logistic regression was used to keep only the significant variables. A significance level of 0.05 is required to allow a variable into the model (SLENTRY = 0.05) and a significance level of 0.05 is required for a variable to stay in the model (SLSTAY = 0.05). No interaction was tested. Hosmer and Lemeshow Goodness-of-Fit test was used to verify that the model was optimal.

Plots of the Kaplan-Meier curves for time to all-cause death in relation to eGFR subgroup were performed. The survival distributions have been compared using the log-rank test. Odds ratios [95% confidence intervals (CI)] comparing the categories of eGFR were derived from a logistic model.

Weighted Cohen’s kappa coefficient^16^ was used to assess the agreement in classification of patients in the different categories of eGFR (≥80 ml/min, between 50 and 79 ml/min, between 30 and 49 ml/min and <30 ml/min) with the 4 different equations used for eGFR.

The relationship between eGFR categories and death prediction was evaluated through the AUCs of the ROC curves and ROC curves were then compared according to De Long, De Long and Clarke-Pearson method^17^.

A two-sided p value of <0.05 was considered as statistically significant. All analyses were performed using SAS statistical software version 9.3 (SAS Institute, Inc., Cary, NC, USA).

## Results

**Figure 1:**
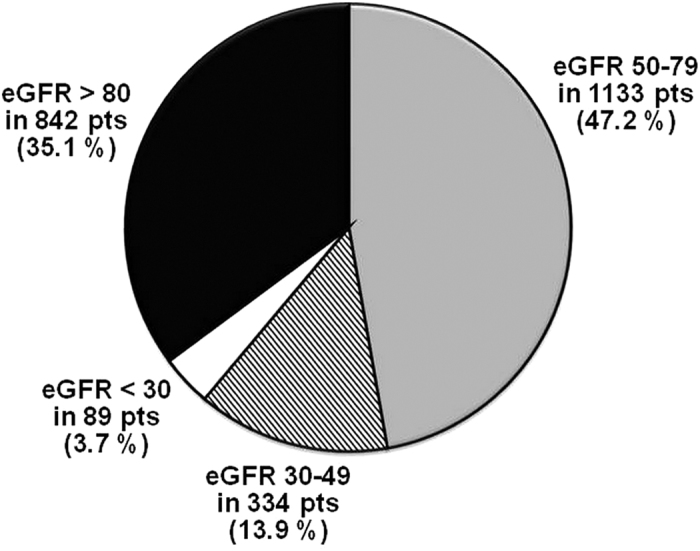
Distribution of patient population according to eGFR calculated with CKD-EPI equation.

A total of 2398 patients were evaluated in the present analysis, on the basis of availability of baseline data for calculating eGFR, as well as availability of 1-year follow-up data or at least information on vital status at 1 year. This population of 2398 patients corresponds to 90.8% of the 2642 subjects with follow-up data. The distribution of patient population according to eGFR calculated with CKD-EPI equation is shown in Fig. 1. Of the study cohort, 47.2% had mild renal impairment, and 17.6% had moderate-severe renal impairment.

### Clinical characteristics associated with different categories of eGFR

The clinical characteristics of enrolled patients according to kidney function, as expressed by eGFR calculated with CKD-EPI, are shown in Table 1. There was a higher prevalence of elderly subjects, heart failure, valvular alterations, diabetes mellitus, hypertension and peripheral artery disease in the lower eGFR groups. Despite this, AF was more frequently asymptomatic (EHRA score I) in patients with severely compromised eGFR.

With regard to thrombotic risk, a history of ischaemic thrombo-embolic complications but not history of prior stroke, was more common in patients with more severely compromised eGFR reflecting the higher mean CHADS_2_ and CHA_2_DS_2_VASc scores. A history of hemorrhagic events, but not major bleeding per se, was more commonly found in patients with severely compromised eGFR and this was associated with higher HAS BLED scores (Table 1).

### Prescribed interventions and medications

Interventions and medications at discharge/after consultation according to stages of renal function are shown in Table 2. There was more use of a rate control strategy in patients with progressively worse renal function. Overall use of antithrombotic drugs differed according to eGFR, with significantly lower prescription at discharge of oral anticoagulants (Table 2). Among AF patients with eGFR <30 ml/min/1.73 m^2^ around 30% did not receive oral anticoagulants.

### Outcomes at 1-year follow up and relationships with eGFR

Outcomes at 1-year follow up according to stages of renal function (eGFR with CKD-EPI equation) are shown in Table 3. A progressive increase in the rate of adverse events observed at 1-yr follow up was found, mainly related to a steep increase in all-cause death in subgroups of patients belonging to categories with lower eGFR. For all-cause death and for the composite end-point of ‘stroke/TIA/death’ the event rate at 1-year was almost 10-fold higher in patients with eGFR below 30 ml/min/1.73 m^2^, as compared to patients with eGFR above 80 ml/min/1.73 m^2^. Readmissions to hospital for cardiac reasons was also common (approximately one third of patients) with limited differences among eGFR subgroups, while readmission for non cardiac reasons was more common when renal function was more compromised.

In order to assess the potential impact of confounding factors on the relationship between eGFR and outcome, different models were considered with variable adjustments. The evolution of odds ratio for from crude models to fully adjusted models is reported in Table 4. As shown, the impact of confounding factors changes the values of odds ratio, but the odds of CKD stages still remains significant in all models.

**Figure 2:**
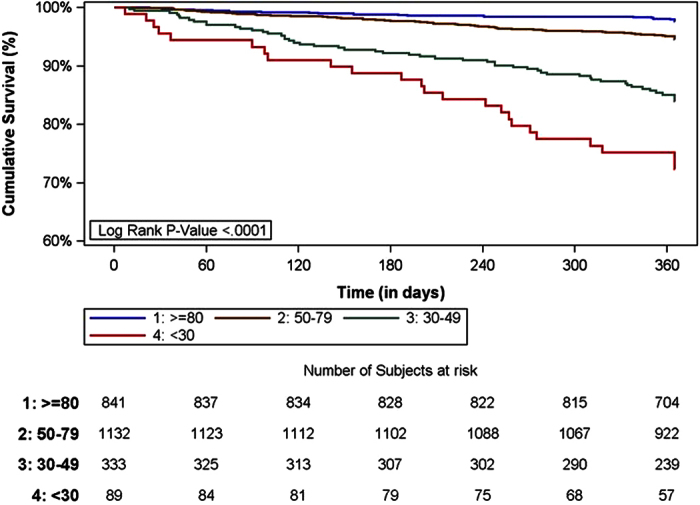
Kaplan Meier curve of freedom from all-cause death according stages of renal function (eGFR with CKD-EPI equation). (Log rank chi-square = 144.88, p < 0.0001).

Kaplan Meier curves of freedom from all-cause death according to different categories of renal function (eGFR with CKD-EPI equation) are shown in Fig. 2. Survival was significantly worse with eGFR below 30 ml/min (Log rank chi-square = 144.88, p < 0.0001).

### eGFR with MDRD, Cockcroft-Gault and adjusted Cockcroft-Gault equations and outcomes

In Table 5 we show other calculations of eGFR using equations based on serum creatinine, and used in clinical practice, that is, MDRD, Cockcroft-Gault, Cockcroft-Gault adjusted for BSA. As expected, some differences were found in the number of patients allocated to each category of eGFR, according to the different formulas.

Using Cohen’s weighted K test for the concordance of attribution to each class of eGFR, we found agreement between the categorisations based on Cockcroft-Gault and Cockcroft-Gault adjusted equations, and those based on either CKD-EPI or MDRD equations were moderate to substantial (weighted K coefficients between 0.5755 and 0.6404)^16^. Agreement between attributions based on CKD-EPI and MDRD was high and could be interpreted as “almost perfect agreement” (weighted K coefficient of 0.8918)^16^.

**Figure 3:**
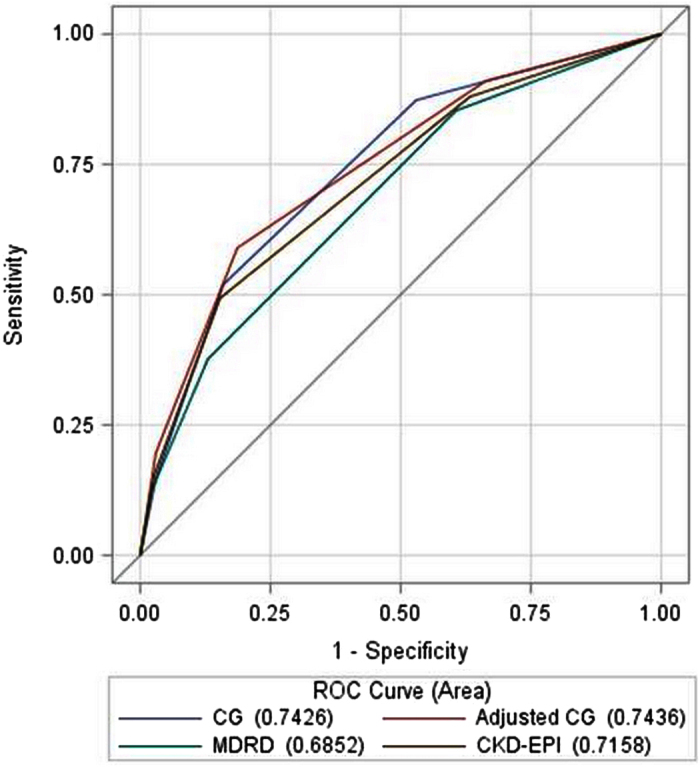
ROC curves and AUCs for death prediction according to eGFR categories with different equations for eGFR.

Kaplan Meier curves of freedom form all-cause death according to different categories of renal function, by calculating eGFR with MDRD, Cockcroft-Gault and Cockcroft-Gault adjusted for BSA equations are shown in the Supplementary web-only material (Figures w1, w2, w3). Irrespective of eGFR equation used, survival at 1 year significantly differed according to eGFR categorisation. According to AUCs of the ROC curves, the best discriminant capability for death prediction according to eGFR categories was found for the Cockcroft-Gault equation adjusted for BSA (p < 0.0001 vs. MDRD equation and p = 0.0238 vs. CKD-EPI equation), followed by Cockcroft-Gault equation (p = 0.0002 vs. MDRD equation and p = 0.0676 vs. CKD-EPI equation), and CKD-EPI equation (p = 0.0023), while the worst was found for the MDRD equation (Fig. 3). No statistically significant difference was found between Cockcroft-Gault equation and the same equation adjusted for BSA (p = 0.9205).

## Discussion

We found that in a group of patients with AF presenting to cardiologists across Europe evaluation of renal function with eGFR has important implications with regard to 1-year outcome (all-cause death or the combined end-point Stroke/TIA/death). Our principal findings indicate that only around 35% of patients have a normal eGFR and that AF is more frequently asymptomatic when it occurs in patients with severely compromised eGFR. Second, with regard to outcomes at 1-year follow up, we found that lower eGFR categories are associated with a steep increase in all-cause death, and with more hospital readmission for non cardiac reasons. Indeed, a reduced eGFR category appears to be a strong independent predictor of the end point of stroke/TIA or death and even mild or moderate impairments in renal function are associated with a significantly increased risk. Third, the concordance between the different equations for estimating GFR was variable, and the best discriminant capability for the risk of death was found for the Cockcroft-Gault equation adjusted for BSA, followed by Cockcroft-Gault equation, and CKD-EPI equation, while the MDRD equation had the worst performance.

EORP-AF is a “real world” cardiology registry exploring all the spectrum of patients with AF presenting to cardiologists, with no exclusions due to characterization of AF as “valvular” or “non valvular”, extent of renal or hepatic dysfunction, age, malignancy or other co-morbidities^10^,^11^,^12^,^13^,^14^,^15^. Only around one third of the patients enrolled in EORP-AF had a normal eGFR (i.e. >80 ml/min/1.73 m^2^) and therefore the finding that any category of eGFR below this normal value is associated with a worse outcome has clinical implications applicable to a large proportion of AF patients.

In our study, significant differences in outcome at 1 year were found between different categories of renal function with regard to all-cause death and the combined end-point of stroke/TIA/death, while limited differences were found in the occurrence of stroke. The percentage of patients treated with oral anticoagulants, according to guidelines was relatively high (around 83–84%), with the only exception seen in patients with eGFR < 30 ml/min/1.73 m^2^, where the use of oral anticoagulants dropped below 70%. The overall high use of anticoagulants may explain the relatively low incidence of strokes in a 1-year follow up period. As shown by Marijon *et al*. with regard to patients with non-valvular AF, the majority of deaths that occur in a contemporary anticoagulated AF population are not related to stroke, which per se only accounted for 7% of the cases of deaths observed^18^.

In EORP-AF, hospitalizations for non cardiac causes markedly increased in relation to decreased renal function and this finding, related to the frequent association between CKD and other co-morbidities, such as hypertension, coronary disease and diabetes mellitus, has important implications in terms of organization of health care (need for a multidisciplinary approach) and cost of care^2^,^19^.

Indeed, even mild to moderate reduction of eGFR have an impact on patients outcome in terms of increased risk of the combined end-point corresponding to TIA/stroke or death at 1 year. Of note, the increase in risk related to renal function can be more appropriately appreciated by considering allocation to categories of eGFR, rather than by considering more generic information such as “history of CKD”.

Our findings emphasise that assessment of eGFR, in daily practice should be done not only for calculating the appropriate dosing of drugs eliminated by the kidney, such as NOACs, but also for identifying those patients who have a worse prognosis and require more strict clinical surveillance. Detection and treatment of associated conditions like uncontrolled hypertension, diabetes or significant coronary disease are mandatory, as well as detection of (micro)albuminuria and assessment by a nephrologist to prevent further deterioration of renal function. Previous registries that investigated the outcome of patients with non valvular AF, including the ATRIA study^20^ and the Loire Valley Atrial Fibrillation Project^21^ did not analyse differences in outcome above a value of eGFR ≥ 60 ml. Our study provides the clinically important information that the risk of death or TIA/stroke/or death at 1 year is elevated even in patients in whom renal function is considered only slightly decreased, i.e. the risk is two-fold for patients with eGFR calculated with CKD-EPI formula between 50 and 79, as compared to patients with eGFR ≥80 ml/min/1.73 m^2^.

In clinical practice, many equations have been proposed for calculating eGFR on the basis of serum creatinine^2^,^9^, but it is unclear what formula is more commonly used or should be used. The KDIGO document recommends use of the CKD-EPI equation^9^, while randomized trials that validated NOACs versus warfarin used the Cockcroft-Gault equation for dosing NOACs through eGFR^2^,^8^,^22^ and many laboratories report eGFR calculated with MDRD formula anytime serum creatinine is measured. In general, as already reported^2^, the clinician should remain aware of caveats for any estimating equation.

The MDRD^23^ equation uses 4 variables (age, gender, serum creatinine and ethnicity) to calculate eGFR and is widely used, especially in routine reports by many laboratories. The CKD-EPI equation^24^, which uses the same 4 variables as the MDRD, is becoming more widely adopted^25^, according to recommendations^9^, and appears to have greater accuracy and precision than the MDRD equation, with less bias at GFR > 60 ml/min/1.73 m^2 ^26. The Cockcroft-Gault equation, proposed around 40 years ago^27^, has a series of bias in patients with a high body weight or BMI and its overall accuracy is lower than that of the two other formulas^28^. In this context, our analysis on the concordance in eGFR values and in allocation to different classes of renal dysfunction according to the different formulas used for eGRF, as done in our analysis, is of clinical value. While the discordance between MDRD, CKD-EPI and Cockcroft-Gault formulas was previously reported by Manzano-Fernandez *et al*.^29^ with regard to dosing of NOACs,no prior analysis has compared these formulas with regard to prognostic implications in AF patients. In our study, the concordance in allocation to eGFR categories was lower when considering the Cockcroft-Gault equation versus the other two equations, similarly to what found in patients with heart failure^30^.

With regard to the risk of death significant differences according to allocation to different eGFR categories were found. Even if the predicting capabilities may show some variations according to the equation used for eGFR calculation, the prediction of worse outcome in the presence of a low eGFR category is maintained for every specific eGFR formula adopted. Of note, in our study, the best discriminant capability for death at 1 year was found for the Cockcroft-Gault equation adjusted for BSA, suggesting that this should perhaps be the method of choice when using eGFR for predicting the risk of death.

### Limitations

The patients were enrolled in EORP AF through cardiology clinics and therefore the study findings cannot be generalized to patients treated by internists, or general practitioners. Albuminuria, which is an important component of assessment of kidney dysfunction was not evaluated, similarly to many other studies performed in the cardiology setting^2^,^21^. Finally, as in any observational study we cannot exclude the presence of some residual confounding, persisting despite adjustment for a series of variables, due to unmeasured factors, or binarily categorized factors.

## Conclusions

In this one-year follow-up analysis of a registry of “real world” patients with AF followed by cardiologists, renal function was a major determinant of adverse outcomes, and even mild or moderate impairments in renal function were associated with an increased risk of stroke/TIA/death. The best discriminant capability for death according to eGFR categories was found for the Cockcroft-Gault equation adjusted for BSA.

## Additional Information

**How to cite this article**: Boriani, G. *et al*. Glomerular filtration rate in patients with atrial fibrillation and 1-year outcomes. *Sci. Rep.* **6**, 30271; doi: 10.1038/srep30271 (2016).

## Supplementary Material

Supplementary Information

## Figures and Tables

**Table 1:** Patient characteristics at enrollment according to stages of renal function (eGFR with CKD-EPI equation).

	All	CKD-EPI ≥80 ml/min/1.73 m^2^	CKD-EPI 50–79 ml/min/1.73 m^2^	CKD-EPI 30–49 ml/min/1.73 m^2^	CKD-EPI <30 ml/min/1.73 m^2^	P value
No. of patients	2398	842	1133	334	89	
Demographics						
Age in years Median (IQR)	69 (62–77)	64 (56–71)	71 (64–78)	76 (69–82)	76 (71–83)	<0.0001
Age > = 75 yrs (%)	32.7	15.8	35.7	57.5	60.7	<0.0001
Age > 65 yrs (%)	63.3	43.3	70.1	85.0	85.4	<0.0001
Female gender (%)	39.3	30.5	41.9	50.9	46.1	<0.0001
AF type (%)						
First detected	30.3	28.5	30.7	31.3	39.8	0.0001
Paroxysmal	26.5	31.2	26.0	19.5	14.8	
Persistent	27.0	26.3	27.2	30.0	20.5	
Permanent	16.1	14.0	16.1	19.2	25.0	
Concomitant disease (%)						
Lone AF	4.1	7.8	2.7	0.3	0	<0.0001
Coronary artery disease	35.7	26.1	38.5	47.8	46.3	<0.0001
Myocardial infarction	44.6	40.3	41.0	55.6	63.2	0.0012
PTCA/CABG	46.8	44.8	45.8	50.0	55.3	0.5372
Stable angina	36.3	37.8	36.6	34.7	31.6	0.8641
Chronic heart failure	46.2	34.7	46.9	64.5	76.4	<0.0001
of whom NYHA III/IV	43.4	39.9	39.8	51.4	60.3	0.0005
Valvular heart disease	61.2	51.4	62.5	77.0	78.8	<0.0001
Dilated cardiomyopathy	12.1	12.3	11.0	12.8	20.7	0.0559
Hypertrophic cardiomyopathy	4.1	4.0	4.2	3.3	5.7	0.7655
Restrictive cardiomyopathy	0.6	0.4	0.5	1.5	1.1	0.0972[*]
Hypertensive cardiomyopathy	19.8	18.2	20.0	22.7	20.7	0.3794
Other cardiac disease	9.0	9.4	7.8	9.7	17.9	0.0156
Chronic obstructive pulmonary disease	12.0	10.8	10.7	18.8	13.8	0.0005
Hyperthyroidism	3.0	4.1	2.5	1.9	2.4	0.1413
Hypothyroidism	7.2	4.1	8.3	8.5	17.6	<0.0001
Chronic kidney disease	13.7	1.0	8.0	45.0	92.0	<0.0001
Peripheral vascular disease	12.2	8.8	11.2	18.9	32.6	<0.0001
Cardiovascular risk factors (%)						
Diabetes	21.3	16.0	20.9	31.6	39.5	<0.0001
Hypertension	71.2	62.5	73.4	80.7	91.0	<0.0001
Current smoker	11.3	16.2	9.2	8.4	3.4	<0.0001
Hypercholesterolaemia	49.1	41.9	52.0	54.9	58.0	<0.0001
Alcohol >= 2–3/day	8.9	11.6	8.5	4.4	4.8	0.0008
Physical activity (%)						
None	38.2	30.0	37.7	53.7	62.8	<0.0001
Occasional	35.4	35.5	36.1	35.1	25.6	
Regular	21.7	28.1	21.5	9.9	9.3	
Intense	4.7	6.4	4.6	1.3	2.3	
Co-morbidities (%)						
Ischaemic thrombo-embolic complications	12.9	10.3	12.2	19.6	22.1	<0.0001
Previous stroke	6.3	5.6	6.1	7.5	9.1	0.4364
Previous Transient Ischaemic Attack	3.9	3.2	3.3	6.9	6.8	0.0071
Haemorrhagic events	6.3	3.8	6.4	7.9	23.9	<0.0001
Haemorrhagic stroke	4.6	3.1	5.6	3.8	4.8	>0.999[*]
Major bleeding	25.8	21.9	26.4	23.1	33.3	0.8029
Malignancy	5.5	4.0	6.1	6.5	7.1	0.1399
Main reason for admission/consultation (%)						
Atrial fibrillation	60.2	70.4	60.8	40.7	28.1	<0.0001
Acute myocardial infarction	4.4	2.9	4.8	7.5	3.4	
Valvular heart disease	3.4	2.1	3.8	5.1	3.4	
Hypertension	1.1	0.8	1.2	1.2	2.2	
Heart failure	17.1	11.9	15.6	29.0	41.6	
Other coronary artery disease	4.2	4.2	4.7	3.6	1.1	
Other cardiac	6.9	5.7	6.3	10.8	12.4	
Other non-cardiac reason	2.6	2.0	2.8	2.1	7.9	
Symptoms						
EHRA I	40.2	34.9	40.7	47.3	57.3	<0.0001
EHRA II	30.9	34.8	30.6	25.1	18.0	
EHRA III–IV	28.9	30.3	28.7	27.5	24.7	
CHADS_2_ score						
Mean score ± SD	1.93 ± 1.27	1.47 ± 1.17	1.97 ± 1.20	2.64 ± 1.24	2.99 ± 1.10	<0.0001
Two or more	60.3	44.1	62.8	84.4	92.1	
CHA_2_DS_2_- VASc score						
Mean score ± SD	3.25 ± 1.80	2.43 ± 1.67	3.36 ± 1.65	4.47 ± 1.65	4.87 ± 1.42	<0.0001
Two or more	81.6	66.5	87.1	96.4	98.9	
HAS-BLED score						
Mean score ± SD	1.37 ± 1.08	0.95 ± 0.96	1.39 ± 0.97	2.00 ± 1.06	2.79 ± 1.07	<0.0001
Two or more	41.1	24.9	41.7	66.8	91.0	

Kruskal-Wallis test is used for quantitative data. Chi-2 or Fisher exact test [*] is used for binary variables. For qualitative variables with more than 2 possibilities, the Monte Carlo estimates of the exact p-values are used. IQR, interquartile range.

**Table 2:** Prescribed interventions and medications at discharge/after consultation according to stages of renal function (eGFR with CKD-EPI equation).

	All	CKD-EPI ≥80 ml/min/1.73 m^2^	CKD-EPI 50–79 ml/min/1.73 m^2^	CKD-EPI 30–49 ml/min/1.73 m^2^	CKD-EPI <30 ml/min/1.73 m^2^	P value
No. of patients	2398	842	1133	334	89	
Management Strategy (%)						
Rate control	38.4	33.4	38.5	45.8	56.2	<0.0001
Rate and rhythm control	44.5	48.3	44.3	40.1	25.8	
Rhythm control only	13.3	15.0	13.6	8.1	12.4	
Observation	3.9	3.3	3.6	6.0	5.6	
Interventions (%)						
(on inpatients only)	1647	570	762	250	65	
Pharmacological cardioversion	29.2	33.0	28.9	25.3	15.6	0.0095
Electrical cardioversion	24.2	25.3	25.5	21.5	9.4	0.0220
Catheter ablation	9.6	15.9	8.3	2.0	0	<0.0001
Pacemaker implantation	4.2	3.5	5.0	3.2	4.6	0.4771
ICD Implantation	1.0	0.9	0.7	2.4	1.5	0.1020[*]
Surgical therapy of AF	0.3	0.5	0.3	0	0	0.6601[*]
Antithrombotic treatments (%)						
None	4.0	6.4	2.7	2.7	2.2	0.0002
Antiplatelets	34.3	29.7	35.3	41.3	39.3	0.0009
Oral anticoagulant	81.4	80.0	82.9	82.6	69.7	0.0104
Vitamin K antagonists	90.4	90.8	88.7	93.5	98.4	0.0127
NOAC	9.7	9.4	11.3	6.5	1.6	0.0134
Oral anticoagulant if indicated**	83.0	82.8	84.3	83.1	69.7	0.0058
of whom Vit K antagonists	90.7	91.2	88.9	93.5	98.4	0.0153
of whom NOAC	9.4	8.9	11.1	6.5	1.6	0.0166
Antiarrhythmic drugs (%)						
At least one	35.4	35.8	37.0	32.1	24.7	0.0633
Amiodarone	21.2	19.4	21.5	24.9	21.3	0.2124
Beta-blockers	70.7	68.4	70.6	76.6	71.9	0.0483
Digoxin	20.1	17.7	21.4	22.5	18.0	0.1372
ACE inhibitors	44.2	41.1	48.2	42.9	27.0	<0.0001
ARBs	21.5	18.8	22.7	26.7	13.5	0.0039
Diuretics	52.7	39.4	54.5	72.5	80.9	<0.0001
Aldosterone blockers	25.5	19.4	26.0	38.6	27.3	<0.0001

NOAC, Non-VKA Oral AntiCoagulant. ACE, angiotensin converting enzyme. ARB, angiotensin receptor blockers. if indicated**, CHA_2_DS_2_-VASc ≥ 2 or pharmacological cardioversion planned. *Chi-2 or Fisher exact test used for binary variables.

**Table 3:** Outcome in terms of adverse events at 1-year follow up according to stages of renal function (eGFR with CKD-EPI equation).

	All	CKD-EPI ≥80 ml/min/1.73 m^2^	CKD-EPI 50–79 ml/min/1.73 m^2^	CKD-EPI 30–49 ml/min/1.73 m^2^	CKD-EPI <30 ml/min/1.73 m^2^	P-value
No. of patients	2398	842	1133	334	89	
Events n(%)						
Stroke/TIA/death	178 (7.9%)	22 (2.8%)	73 (7.0%)	58 (18.5%)	25 (28.7%)	<0.0001
All cause death	156 (6.5%)	19 (2.3%)	60 (5.3%)	53 (15.9%)	24 (27.0%)	<0.0001
Stroke/TIA	22 (1.1%)	3 (0.4%)	13 (1.3%)	5 (1.9%)	1 (1.6%)	0.0509[*]
Bleeding	23 (1.1%)	6 (0.8%)	10 (1.0%)	6 (2.3%)	1 (1.6%)	0.1598[*]
Re-admission for cardiac reason	592 (28.6%)	196 (25.5%)	287 (29.4%)	91 (34.9%)	18 (29.5%)	0.0283
Re-admission for non cardiac reason	268 (12.8%)	84 (10.8%)	125 (12.6%)	40 (15.4%)	19 (30.6%)	<0.0001

Any TE = Stroke, TIA, ACS, Coronary intervention, cardiac arrest, peripheral embolism and pulmonary embolism. *Fisher exact test used for binary variables.

**Table 4:** Evolution of odds ratio [95% Wald Confidence Limits] of eGFR with the outcome stroke/TIA or death at 1 year.

Model	eGFR CKD-EPI <30 vs ≥80 ml/min/1.73 m^2^	eGFR CKD-EPI 30–49 vs ≥80 ml/min/1.73 m^2^	eGFR CKD-EPI 50–79 vs ≥80 ml/min/1.73 m^2^	Overall Pr > ChiSq
Model 1 (a)	7.955 [4.104; 15.418] p < 0.0001	4.622 [2.680; 7.972] p < 0.0001	1.883 [1.142; 3.105] p = 0.0132	p < 0.0001
Model 2 (b)	6.834 [3.248; 14.377] p < 0.0001	4.185 [2.260; 7.749] p < 0.0001	1.912 [1.083; 3.376] p = 0.0255	p < 0.0001
Model 3(c)	5.616 [2.258; 13.971] P = 0.0002	4.135 [1.902; 8.991] P = 0.0003	2.247 [1.117; 4.520] p = 0.0231	P = 0.0004
Model 4 (d)	4.699 [2.113; 10.449] P = 0.0001	3.004 [1.551; 5.819] P = 0.0011	1.792 [0.978; 3.285] p = 0.0591	P = 0.0003

(**a**) Model 1 includes age and sex. Hosmer and Lemeshow Goodness-of-Fit p-value = 0.2248. (**b**) Model 2 includes age, sex and co-morbidities: coronary artery disease, chronic heart failure, previous stroke, previous TIA, ischaemic thrombo-embolic complications, haemorrhagic events and malignancy. Hosmer and Lemeshow Goodness-of-Fit p-value = 0.1183. (**c**) Model 3 is the model 2 with confounding factors significant (p < 0.1) in the univariate model, i.e. nation, setting, AF type, valvular heart disease, cardiomyopathy, other cardiac disease, COPD, peripheral vascular disease, diabetes, alcohol >= 2–3/day, physical activity, previous pharmacological cardioversion, electrical cardioversion, catheter ablation, EHRA symptoms - BMI - SBP - DBP - Management Strategy - Main reason for admission/consultation. Hosmer and Lemeshow Goodness-of-Fit p-value = 0.7486. (**d**) Model 4 is the model 2 with confounding factors significant (p < 0.1) in the univariate model and kept into the model with the stepwise procedure, i.e. nation, diabetes, physical activity and main reason for admission/consultation. Hosmer and Lemeshow Goodness-of-Fit p-value = 0.6485.

**Table 5:** Evaluation of eGFR with different equations (top panel) and concordance of attribution to each class of eGFR with Cohen’s weighted K test (bottom panel).

	CKD-EPI (ml/min/1.73 m^2^)	MDRD (ml/min/1.73 m^2^)	Cockcroft-Gault (ml/min)	Cockcroft-Gault adjusted (ml/min/1.73 m^2^)
	2398 patients	2398 patients	2398 patients	2398 patients
Number of patients (%) with eGFR ≥80	842 (35.1%)	902 (37.6%)	1076 (44.9%)	770 (32.1%)
Number of patients (%) with eGFR 50–79	1133 (47.2%)	1143 (47.7%)	879 (36.7%)	1117 (46.6%)
Number of patients (%) with eGFR 30–49	334 (13.9%)	285 (11.9%)	354 (14.8%)	411 (17.1%)
Number of patients (%) with eGFR <30	89 (3.7%)	68 (2.8%)	89 (3.7%)	100 (4.2%)
Mean ± SD of eGFR	69.93 ± 21.19	73.77 ± 23.87	79.74 ± 33.77	70.41 ± 25.71
**Weighted K coefficients (and 95% CI) and classes of renal function based on eGFR estimates**				
	**MDRD**	**Cockcroft-Gault**	**Cockcroft-Gault adjusted**	
**CKD-EPI**	0.8918 (0.8776–0.9061)	0.6242 (0.6000–0.6483)	0.7254 (0.7039–0.7469)	
**MDRD**		0.5755 (0.5499–0.6012)	0.6404 (0.6164–0.6643)	
**Cockcroft-Gault**			0.7654 (0.7460–0.7848)	
