# Corrigendum: Glomerular filtration rate in patients with atrial fibrillation and 1-year outcomes

**DOI:** 10.1038/srep42379

**Published:** 2017-02-22

**Authors:** Giuseppe Boriani, Cécile Laroche, Igor Diemberger, Mircea Ioachim Popescu, Lars Hvilsted Rasmussen, Lucian Petrescu, Harry J. G. M. Crijns, Luigi Tavazzi, Aldo P. Maggioni, Gregory Y. H. Lip

Scientific Reports 6: Article number: 3027110.1038/srep30271; published online: 07 28 2016; updated: 02 22 2017

This Article contains errors in the Acknowledgements section:

“Abbott Vascular Int. (2011–2014), Amgen (2012–2018), AstraZeneca (2014–2017), Bayer (2013–2018), Boehringer Ingelheim (2013–2016), Boston Scientific (2010–2012), The Bristol Myers Squibb and Pfizer Alliance (2014–2016), The Alliance Daiichi Sankyo Europe GmbH and Eli Lilly and Company (2014–2017), Gedeon Richter Plc. (2014–2017), Menarini Int. Op. (2010–2012), MSD-Merck & Co. (2011–2014), Novartis Pharma AG (2014–2017), ResMed (2014–2016), Sanofi (2010–2011), SERVIER (2012–2018)”.

should read:

“Abbott Vascular Int. (2011–2014), Amgen (2009–2018), AstraZeneca (2014–2017), Bayer (2009–2018), Boehringer Ingelheim (2009–2016), Boston Scientific (2009–2012), The Bristol Myers Squibb and Pfizer Alliance (2011–2016), The Alliance Daiichi Sankyo Europe GmbH and Eli Lilly and Company (2011–2017), Gedeon Richter Plc. (2014–2017), Menarini Int. Op. (2009–2012), MSD-Merck & Co. (2011–2014), Novartis Pharma AG (2014–2017), ResMed (2014–2016), Sanofi (2009–2011), SERVIER (2009–2018)”.

