# Supplementary material for: Glomerular filtration rate in patients with atrial fibrillation and 1-year outcomes
Source: Sci Rep. 2016 Jul 28;6:30271. doi: 10.1038/srep30271 (PMC4964613; doi:10.1038/srep30271)
Supplement: Supplementary Information [file srep30271-s1.doc]

**Supplementary Web-only material**

**Glomerular filtration rate in patients with atrial fibrillation**

**and 1-year outcomes**

***The EURObservational Research Programme - Atrial Fibrillation General Registry Pilot Phase (EORP-AF Pilot registry)***

Giuseppe Boriani^1, 2^, Cécile Laroche^3^, Igor Diemberger^2^, Mircea Ioachim Popescu^4^,

Lars Hvilsted Rasmussen5, Lucian Petrescu^6^, Harry JGM Crijns^7^, Luigi Tavazzi^3, 8^,

Aldo P Maggioni^3,9^, Gregory Y H Lip^10 *^

Affiliations:

1 Cardiology Department, University of Modena and Reggio Emilia, Policlinico di Modena, Modena, Italy

2 Institute of Cardiology, Department of Experimental, Diagnostic and Specialty Medicine, University of Bologna, S.Orsola-Malpighi University Hospital, Bologna, Italy

3 EURObservational Research Programme Department, European Society of Cardiology, Sophia Antipolis, France

4 Faculty of Medicine, Cardiology Department, Oradea, Romania

5 Department of Cardiology, Aalborg University Hospital and Aalborg Thrombosis Research Unit, Department of Clinical Medicine, Faculty of Medicine Aalborg University, Aalborg, Denmark

6 Institute of Cardio-vascular Diseases, University of Medicine and Pharmacy "Victor Babes", Timisoara, Romania

7Department of Cardiology and Cardiovascular Research Institute Maastricht (CARIM), Maastricht University Medical Center, The Netherlands

8 Maria Cecilia Hospital, GVM Care&Research . E.S. Health Science Foundation, Cotignola, Italy

9 ANMCO Research Center, Firenze Italy

10 University of Birmingham Centre for Cardiovascular Sciences, City Hospital, Birmingham B18 7QH, United Kingdom

* The complete list is in this Supplementary Appendix .

**Table w1.** Equations proposed for estimated glomerular filtration rate (eGFR).

**Cockcroft-Gault (CG) equation:**

If creatinine in μmol/l,

eGFR CG= (140-age) x weight (kg) x constant/serum creatinine in μmol/l, constant=1.04 if female and 1.23 if male.

If creatinine in mg/dl (as in some countries):

eGFR CG= CG= (140-age) x weight (kg) x [0.85 if female] /serum creatinine in mg/dl x 72

In general (also for the other equations) Creatinine levels in µmol/L can be converted to mg/dL by dividing them by 88.4

**Cockcroft-Gault (CG) equation BSA adjusted (CG adjusted)** :

eGFR CG adj = The same CG formula normalized for a BSA of 1.73 m2 (BSA according to Dubois and Dubois

BSA = (W ^0.425^ x H ^0.725^) x 0.007184) so

eGFR CG adj= CG x 1.73m2/BSA

**MDRD equation:**

eGFR MDRD = 186 x [serum creatinine in μmol/l x 0.011] ^(-1.154)^ x age ^(-0.203)^ x [0.742 if female] .

**CKD Epidemiology Collaboration (CKD-EPI) equation:**

eGFR CKD-EPI

for Female: 141 x min (serum creatinine in mg/dl/0.7) ^(-0.329)^ x max (serum creatinine in mg/dl/0.7) ^(-1.209)^ x 0.993^Age^ × 1.018.

for Male: 141 x min (serum creatinine in mg/dl/0.9) ^(-0.411)^ x max (serum creatinine in mg/dl/0.9) ^(-1.209)^ x 0.993^Age^.

**Figure w1. Kaplan Meier curve of freedom form all-cause death according stages of renal function (eGFR with MDRD equation).** Log rank chi-square = 108.82, p< 0.0001.

**
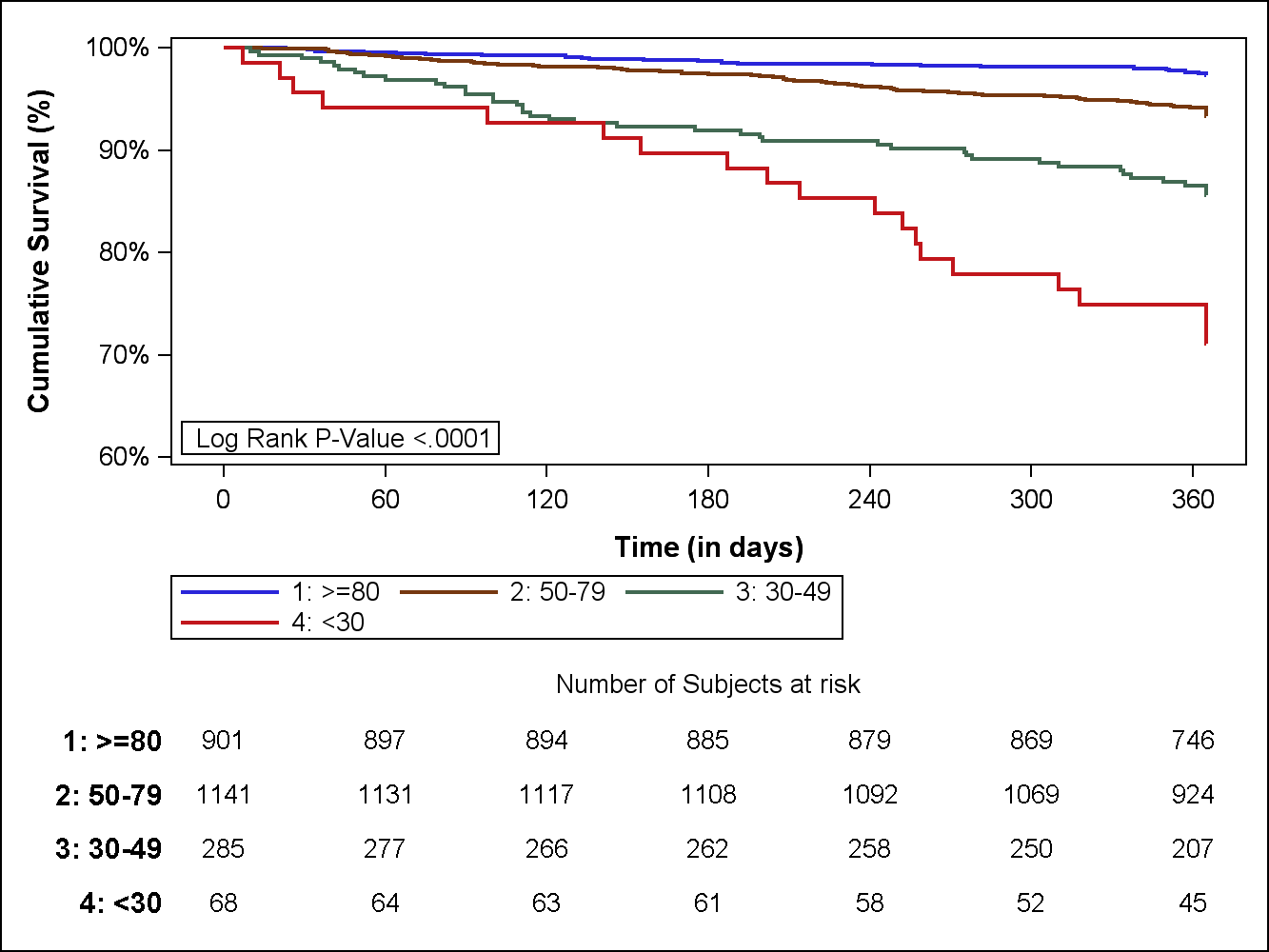
**

**Figure w2. Kaplan Meier curve of freedom form all-cause death according stages of renal function (eGFR with Cockcroft-Gault equation).**.Log rank chi-square = 154.60, p< 0.0001.


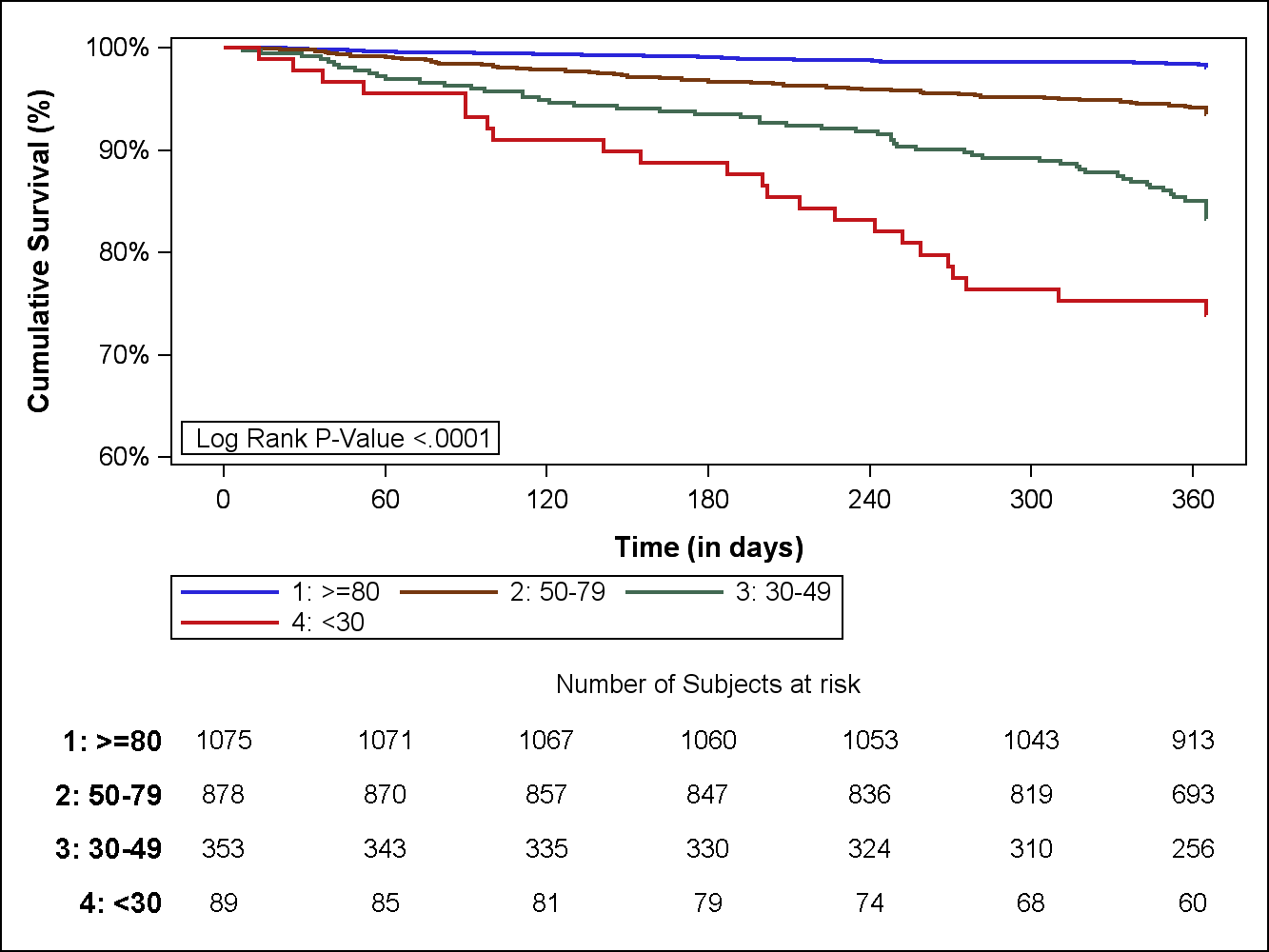


**Figure w3. Kaplan Meier curve of freedom form all-cause death according stages of renal function (eGFR with Cockcroft-Gault equation adjusted for BSA).** Log rank chi-square = 192.88, p< 0.0001.

**
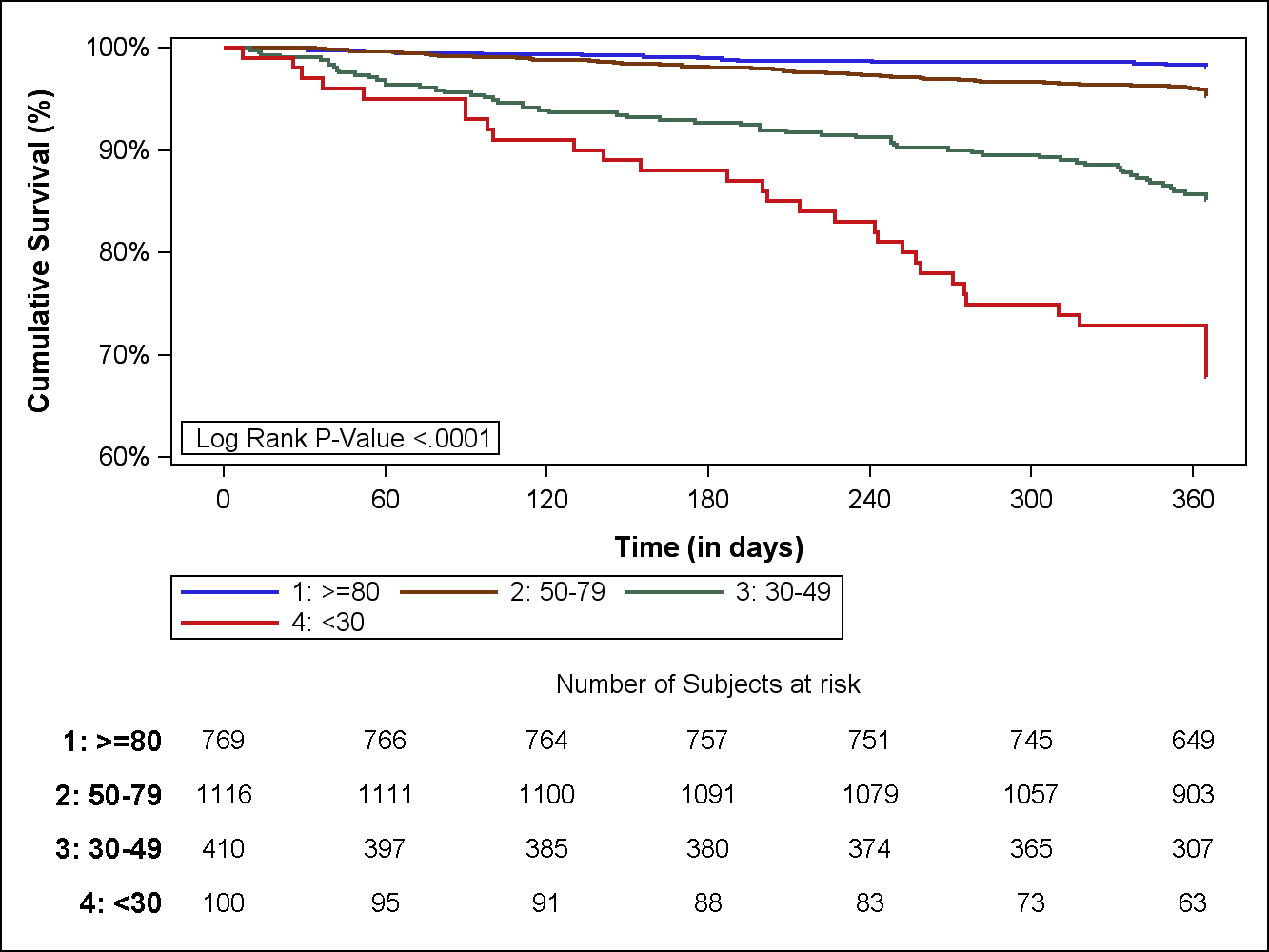
**

**List of EORP Investigators , per country.**

BELGIUM **Bastogne**: M. Raepers, Z. el Husseini; **Hasselt**: D. Dilling-Boer, J. Schurmans, J. Vijgen, P. Koopman; **Wilrijk:** W. Huybrechts; **Yvoir**: F. Dormal, D. Blommaert, O. Deceuninck, O. Xhaet; DENMARK **Aalborg:** C. Fragtrup Hellum, B. Mortensen, B. Ginnerup Sorensen, A. M. Joensen, L. H. Rasmussen; **Copenhagen:** A. Karlsdottir, S. Pehrson; **Esbjerg:** J. Hummelshoj, A-M. Svenningsen, L. Tanggaard, P.Wiggers, A. Nygaard; **Hjorring:** A. Jonstrup, J. Petersen; **Silkeborg:** A. Odgaard, M. Mortensen, L. Frost; **Viborg:** D. Svenstrup Møller, H.M. Søndergaard, P. D. Christensen; GREECE **Athens:** S. Xydonas, L. Lioni; **Chios:** M. Dimopoulou, G. Georgiopoulos, E. Papatheodorou, P. Boutas, A. Kartalis; **Heraklion:** P. Vardas, H. Nakou, E. Kanoupakis, E. Simantirakis; **Thessaloniki:** D. Tahmatzidis, I. Styliadis, V. Vassilikos; **Thessaloniki:** K. Koskinas, N. Fragakis; **Thessaloniki:** K. Polymeropoulos, G. Maligos; ITALY **Bologna:** C. Martignani, I. Diemberger, G. Boriani, J. Frisoni, M. Biffi, M. Ziacchi, P. Cimaglia, E. Fantecchi; **Firenze:** S. Boni, D. Gabbai, N. Marchionni, S. Fumagalli; **Trieste:** M. Bobbo, F.

Ramani, G. Sinagra, L. Vitali-Serdoz, A. Nordio, A. Porto, M. Zecchin, C. Di Nora; NORWAY **Haugesund:** R. Rød, R.M.O. Stødle; **Lorenskog:** M.O. Pervez, P. Smith, M. Buvarp; **Nesttun:**

P.K. Rønnevik; **Oslo:** A. Vold, J. Fuglestved, D. Atar; **Skedsmokorset:** E. Stenshjemmet, K. Risberg; POLAND **Cieszyn:** A. Sokal, A. Kubicius, E. Prochniewicz, K. Pokrywa; **Gorzow:** R. Rzeuski, A.Weryszko; **Katowice:** M. Haberka, Z. Gasior, A. Slowikowski; **Kielce:**M. Janion, M. Kołodziej, A. Janion-Sadowska; **Lodz:** J. Drozdz˙, M. Stasiak, P. Jakubowski, T. Ciurus; **Lodz:** M. Pawlak, M. Nowakowska, K. Wiklo, M. Kurpesa; **Nysa:** A. Olejnik, J. Miarka; **Radlin:** W. Streb; **Warszawa:** L. Zielinski, M. Dluzniewski, M. Tomaszewska- Kiecana; **Warszawa:** G. Opolski, M. Budnik, M. Kiliszek; **Warszawa:** J. Gorska, A. Mamcarz, D. Sliz, K. Makowiecki;**Wroclaw:** A. Fuglewicz, M. Drozd, M. Garncarek; **Zabrze:** A. Musialik-Lydka, E. Markowicz-Pawlus, G. Kazmierczak; **Zabrze:** A. Leopold-Jadczyk, M. Koziel, Z. Kalarus; PORTUGAL **Almada:** S. Sobral, H. Pereira, L. Brandao Alves, L. Ribeiro, R. Miranda, S. Almeida; **Amadora:** F. Madeira, M. Faustino, R. Oliveira, V. Gil; **Braga:** C. Braga, J. Martins, S. Rocha, S. Magalhaes, V. Ramos; **Carnaxide:** R. Bernardo, F. Costa, F. Morgado, P. Galvao Santos, N. Almeida, P. Adragao, P. Carmo; **Coimbra:** G. Mariano Pego, J. Ferreira, L. Elvas, M.Ventura, N. Antonio, R. Ferreira; **Evora:** A.F. Damasio, A.R. Santos, B. Picarra, D. Neves; **Faro:** I.DeJesus, J. Amado, P. Sousa, R. Candeias; **Guimaraes:** A. Lourenco, A. Pereira, F. Canario-Almeida, M. Fernandes, F. Ferreira, I. Machado, I. Quelhas, J. Guardado, V. Pereira; **Lisboa:** D. Cavaco, N. Almeida, P. Adragao, P. Carmo; **Lisboa:** A. Lousinha, B. Valente, N. Silva, P. Cunha, R. Pimenta, S. Santos, M.Martins Oliveira; **Lisboa:** S. Vicente, A. Bernardes, A. Nunes Diogo, E. Rodrigues, J.M. Frazao Rodrigues de Sousa, L. Carpinteiro, M. Satendra, N. Cortez Dias, S. Neto; **Vila Nova de Gaia:** V. Gama Ribeiro, H. Goncalves, J. Primo, L. Adao, M. Oliveira; **Viseu:** A. Costa, A. Delgado, B. Marmelo, D. Moreira, J. Santos, L. Santos, B. Rodrigues; ROMANIA **Arad:** A. Pop Moldovan, D. Darabantiu; **Baia Mare:** B.Todea, C. Pop, D. Dicu, D. Filip, D. Mercea, G. Kozma, M. Schiopu; **Brasov:** G. Catanescu, C. Popescu, E. Bobescu, A. Gabor; **Bucharest:** A. Buzea, A. Dan, I. Daha, N. Asan, R. Popescu, G-A. Dan; **Bucharest:** D. Bartos, E. Badila, E. Tintea, C. Grigore, A.M. Daraban; **Bucharest:** A. Sandulescu, A. Carp, D. Gherasim, I.M. Stoian; **Bucharest:** M.M. Baluta; **Bucharest:** M.M. Vintila; **Oradea:** M.I. Popescu, O. Tica; **Timisoara:** L. Petrescu, N. Alina- Ramona, R. Dan; **Timisoara:** C.D. Constantin, C. Tutuianu, M. Mangea, E. Goanta; THE NETHERLANDS **Enschede:** J. M. van Opstal, R. van Rennes; **Groningen:** B.A. Mulder; **Hengelo:** S. A.M. Said; **Leeuwarden:** R. J. Folkeringa; **Maastricht:** S. Philippens, H.J.G.M. Crijns, Y. Blaauw, I. Aksoy, M. Pluymen, R. Driessen, I. Limantoro, T. Lankveld, M. Mafi Rad, J. Hendriks;**Venlo:**W. H. van Unen, J. Meeder.
